# Haemocompatibility and ion exchange capability of nanocellulose polypyrrole membranes intended for blood purification

**DOI:** 10.1098/rsif.2012.0019

**Published:** 2012-02-01

**Authors:** Natalia Ferraz, Daniel O. Carlsson, Jaan Hong, Rolf Larsson, Bengt Fellström, Leif Nyholm, Maria Strømme, Albert Mihranyan

**Affiliations:** 1 Nanotechnology and Functional Materials, Department of Engineering Sciences, Uppsala University, The Ångström Laboratory, Box 534, 75121 Uppsala, Sweden; 2 Department of Immunology, Genetics and Pathology, Uppsala University, The Rudbeck Laboratory, 75185 Uppsala, Sweden; 3 Renal Medicine, Department of Medical Sciences, Uppsala University Hospital, Akademiska sjukhuset, 75185 Uppsala, Sweden; 4 Department of Materials Chemistry, Uppsala University, The Ångström Laboratory, Box 538, 75121 Uppsala, Sweden

**Keywords:** nanocellulose, polypyrrole, haemodialysis, biocompatibility, heparin coating

## Abstract

Composites of nanocellulose and the conductive polymer polypyrrole (PPy) are presented as candidates for a new generation of haemodialysis membranes. The composites may combine active ion exchange with passive ultrafiltration, and the large surface area (about 80 m^2^ g^−1^) could potentially provide compact dialysers. Herein, the haemocompatibility of the novel membranes and the feasibility of effectively removing small uraemic toxins by potential-controlled ion exchange were studied. The thrombogenic properties of the composites were improved by applying a stable heparin coating. In terms of platelet adhesion and thrombin generation, the composites were comparable with haemocompatible polymer polysulphone, and regarding complement activation, the composites were more biocompatible than commercially available membranes. It was possible to extract phosphate and oxalate ions from solutions with physiological pH and the same tonicity as that of the blood. The exchange capacity of the materials was found to be 600 ± 26 and 706 ± 31 μmol g^−1^ in a 0.1 M solution (pH 7.4) and in an isotonic solution of phosphate, respectively. The corresponding values with oxalate were 523 ± 5 in a 0.1 M solution (pH 7.4) and 610 ± 1 μmol g^−1^ in an isotonic solution. The heparinized PPy–cellulose composite is consequently a promising haemodialysis material, with respect to both potential-controlled extraction of small uraemic toxins and haemocompatibility.

## 1. Introduction

Haemodialysis remains the only treatment for patients with chronic renal failure, who otherwise require kidney transplantation. The normal kidney functions include (i) filtration, i.e. passive removal of metabolic toxins; (ii) reabsorption, i.e. re-uptake of useful substances such as water, glucose and minerals; and (iii) secretion, i.e. active transport of metabolites from peritubular capillaries. Therefore, haemodialysis substitutes only a small fraction of normal kidney function and cannot mimic the metabolic, endocrine and immune functions of the tubule system [1]. The treatments are time-consuming and expensive, as they require patients to attend treatment centres three times a week, for periods of 3–5 h under the supervision of specialized personnel [2]. Furthermore, each treatment requires about 120 l of highly purified water. The increasing prevalence of kidney failure, and the shortage of organ donors further stress the need for innovative approaches for developing haemodialysis membranes and dialysis technology that resembles normal renal function.

When the function of the kidneys fails, uraemic toxins (which are normally excreted) are retained. Uraemic toxins can be subdivided into the following groups: small water-soluble compounds (MW < 500 Da, prototype urea), middle-sized molecules (MW > 500 Da, prototype β2-microglobulin) and protein-bound solutes [3]. Many uraemic toxins belong to the middle-sized molecules and are difficult to remove by dialysis. The failure to remove the middle-sized uraemic toxins has been related to cardiovascular and immunological complications [4]. It is important to retain proteins such as albumin, immunoglobulins and peptide hormones because many are essential, and losses are associated with a great risk for the patient [5]. Therefore, one major challenge for the development of haemodialysis membranes is to selectively remove intermediate molecular weight (toxic) proteins in the size range of 5–35 kDa and avoid the elimination of large molecular weight essential solutes [6].

The recent trend in the development of haemodialysis systems has been to improve the ultrafiltration properties of the membranes by increasing the pore size of the membrane in order to maximize the removal of low molecular weight (toxic) proteins and to enlarge the molecular weight spectrum of solutes removed during dialysis treatments [1]. However, such non-specific strategies also tend to eliminate beneficial solutes such as trace metals or medications [7].

It is also imperative to stress that the complications associated with elevated levels of small size uraemic toxins, such as hyperphosphataemia, hyperhomocysteinaemia or hyperoxalaemia, can lead to serious cardiovascular and immunological problems and eventually death [4,8]. Most patients with end-stage renal failure have a predisposition towards elevated levels of serum phosphorus as glomerular filtration decreases. Even with high-efficiency, high-flux membranes, haemodialysis fails in completely removing the dietary load of phosphorus [9].

In the current work, we present a novel conductive cellulose composite membrane that has the potential of being part of a new generation of haemodialysis systems. The composite consists of nanofibrous cellulose coated with an approximately 50 nm thick layer of the conductive polymer polypyrrole (PPy) [10] and has previously been evaluated for DNA extraction [11] and electric energy storage [12,13].

The composite in the form of a paper sheet has a large surface area (about 80 m^2^ g^−1^) and extensive porosity (about 70%) and can be used for passive filtration, considering that pristine *Cladophora* cellulose has been used as a filter medium in the past [14]. However, the greatest advantage of using this composite material is the possibility to combine ultrafiltration with the electrochemical potential-controlled ion exchange properties of PPy, which are described in detail elsewhere [15]. Briefly, when a sufficiently positive potential is applied, PPy is oxidized, resulting in positively charged polymer chains and small, mobile electrolyte anions move into the bulk material to maintain charge neutrality. When a sufficiently negative potential is applied, the polymer is reduced and anions are released back to the electrolyte solution [16]. It is also possible to introduce cation exchange properties by immobilizing large anions inside the PPy film, as well as to introduce specific ligands capable of highly specific ion recognition and separation [17–19].

Active ion exchange in response to an external electrical stimulus appears highly appealing for removing solutes, and if necessary releasing medicaments, in haemodialysis and other extracorporeal blood treatments. In contrast to conventional electrodialysis, which separates flowing ions in an electric field through a semi-permeable membrane, the PPy ion exchange directly incorporates ions inside the structure. Moreover, by varying the synthesis conditions (e.g. oxidizing agent), it may be possible to vary the network spacing between the conductive polymer chains and, thus, promote the adsorption of low molecular size proteins while leaving large proteins unaffected [20]. Therefore, the properties of the composite material could potentially be tailored to combine active ion exchange and passive diffusion and ultrafiltration through the porous matrix. The exchange process in small liquid volumes will be fast, favouring substantial reduction of the haemodialysis sessions. Moreover, the large surface area of the composite material might lead to a new generation of compact dialysers.

An important requirement for dialysis membranes is haemocompatibility. Blood interaction with the haemodialysis membranes leads to a series of interlinked events such as protein adsorption, platelet and leucocyte adhesion/activation, complement system activation and activation of the coagulation cascade [21]. The activation of circulating blood leucocytes and platelets leads to upregulation of adhesion receptors and release of active species such as cytokines, growth factors and activator factors which in turn can promote further cell activation and adhesion. The complement system plays a central role in leucocyte activation and in the establishment of an inflammatory state [22]. In the chronic haemodialysis patient, these interactions are repetitive, and even mild interactions may lead to adverse clinical consequences, such as haematological changes in the patient blood status (e.g. leucopaenia) and immunological dysfunction [23]. Currently available haemodialysis membranes not only present different physicochemical properties such as performance, pore size and adsorptive capacities, but also show different grades of haemocompatibility. The reason for this is not only differences in chemical composition, but also in the surface roughness, manufacturing conditions and sterilization techniques [1,24].

Several studies have shown the non-cytotoxic nature and good biocompatibility of PPy and its derivatives when tested with a wide number of cell types [25,26]. Studies by Mao *et al*. [27] and Zhang *et al*. [28] highlighted the good blood compatibility of PPy-based materials. The aims of the present work were to investigate the haemocompatibility of a novel PPy–nanocellulose composite material and to study the feasibility of effective removal of small size model uraemic toxins, phosphate and oxalate, by potential-controlled ion exchange.

## 2. Material and methods

### 2.1. Preparation of PPy–cellulose composites

The PPy–cellulose composites were prepared as previously described [26]. Briefly, chemical polymerization of pyrrole on *Cladophora* sp. algae cellulose fibres was carried out with FeCl_3_ as the oxidizing agent. The product was thoroughly rinsed with 35 l of deionized water, followed by 5 l of 0.1 M NaCl at a rate of 7 l h^−1^. After the rinsing step, 8 g of 3 per cent (w/v) microfibrillated cellulose (MFC) was added, and the mixture was ultrasonicated for 1 min. The product was then filtered and dried to obtain 1–2 mm thin composite sheets.

A set of lightly rinsed composite samples, rinsed with 10 l of 0.1 M NaCl, was also prepared for the feasibility studies.

The composite materials were finally incubated with Hank's balanced salt solution (HBSS) for 48 h at room temperature.

### 2.2. Heparinization

In the present work, the Corline method (Corline Systems AB, Uppsala, Sweden) was used to heparin coat the composite materials, the blood collecting tubes and the slide chambers. The Corline heparin-coating process includes a conditioning layer of a polymeric amine (PAV, proprietary agent Corline, Sweden), onto which a macromolecular heparin conjugate is attached by multiple ionic interactions. In the heparin conjugate, approximately 70 heparin molecules are covalently linked to a polymeric carrier. Thereby, the antithrombin (AT)-binding sequence is left intact to interact with AT [29]. The heparin coating applied to the composite materials was a modification of the Corline heparin surface. The composite material is positively charged (PPy is in its oxidized state), and therefore the conditioning layer of the PAV is not needed.

For that reason, the protocol used to heparin-coat the composite surface consisted of the application of the heparin conjugate, followed by an incubation step with PAV and a second layer of heparin conjugate.

The heparinization of the composite membranes was evaluated by the AT binding assay (see below) and by fluorescence microscopy. The heparinized composite membranes were repeatedly reduced and oxidized by cyclic voltammetry (CV) to evaluate the stability of the heparin coating. The effect of the composite steam sterilization by autoclaving (1.5 MPa for 20 min) on the heparin coating was studied by means of the AT binding assay.

#### 2.2.1. Antithrombin binding assay

The AT binding capacity refers to the ability of heparin to interact with the coagulation regulator AT. As a result of the heparin–AT interaction, AT adopts a more active state, increasing its ability of inhibiting different coagulation factors [30]. The AT binding capacity of the heparin-modified composites was evaluated as described by Kodama *et al*. [31]. This method is based on the inhibition of factor Xa by AT in the presence of heparin. The heparin-coated membranes were pre-soaked in phosphate-buffered saline (PBS) and incubated with normal citrate plasma. The membranes were then rinsed with Tris–NaCl (50 mM Tris–HCl, pH 7.8, 0.15 M NaCl) followed by the elution of AT by Tris–NaCl containing 150 IU ml^−1^ heparin (Leo Pharma A/S, Ballerup, Denmark). The AT activity of the eluate was determined in a factor Xa assay. Factor Xa (Chromogenix, Mölndal, Sweden) was added to the sample, incubated for 5 min, followed by the addition of the chromogenic substrate S-2765 (Chromogenix). After 5 min, the reaction was terminated by the addition of citric acid and the remaining factor Xa activity was measured at 405 nm. A calibration curve with normal citrate plasma was used to determine the concentration of AT. The results were expressed as picomoles of AT per centimetre square.

#### 2.2.2. Fluorescence microscopy

The heparin coating was visualized by fluorescence microscopy. The heparinized composite membranes were incubated with avidin conjugated with Texas Red-X according to the kit from Molecular Probes (Invitrogen, Eugene, OR, USA) diluted to 15 µg ml^−1^ in saline solution for 30 min, followed by a washing step with deionized water. The rationale for using avidin–Texas Red is that avidin binds to heparin owing to heparin binding domains in avidin. Samples were observed under a fluorescence microscope (Nikon Eclipse E600, Tokyo, Japan).

### 2.3. Potential-controlled ion exchange

CV was performed in a three-electrode setup using the composite samples as the working electrode, an Ag/AgCl reference electrode and a platinum wire counter electrode. The dried composite samples were attached to a platinum wire sample holder. For each sample, 10 cycles were performed, each including reduction followed by oxidation. The scan rate was 5 mV s^−1^ and the experiments were performed with an Autolab potentiostat (Eco Chemie, The Netherlands).

#### 2.3.1. Stability of heparin coating

The heparin coating stability during electrochemical cycling was studied using heparinized samples which were cycled in 2 M NaCl between −0.6 V and 0.6 V versus Ag/AgCl. The samples were thereafter dried and analysed with the AT binding assay and fluorescence microscopy, as described earlier.

#### 2.3.2. Feasibility study

Non-heparinized and heparinized samples were CV-cycled between −0.8 V and greater than or equal to 0.3 V versus Ag/AgCl; the anodic turning potential was adjusted for each sample to result in a complete oxidation peak and to avoid overoxidation [32]. The samples were cycled in pure 0.10 M oxalate (Na_2_C_2_O_4_, BDH Prolabo) and phosphate (NaH_2_PO_4_, BDH Prolabo) solutions, adjusted to pH 7.4 with NaOH. Samples were also cycled in pure isotonic solutions of oxalate (0.12 M, pH 7.3) and phosphate (0.15 M KH_2_PO_4_, pH 4.4; Sigma-Aldrich, Inc., St Louis, MO, USA). Charge capacities, *Q* (expressed as Coulomb per gram of sample), were calculated from the 10th scan for each sample, by integrating the current over time from the first point of positive current to the current minima located at a more positive potential than the oxidation peak (before overoxidation becomes significant; see arrows in figure 2). The approximate ion exchange capacity (mol g^−1^ sample) was calculated from the charge capacity, assuming the same distribution of differently charged anions in the material as in the electrolyte. The distribution was calculated from the Henderson–Hasselbalch equation, as shown in equation (2.1).2.1
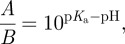
where *A* corresponds to the acid, and *B* the base of the acid–base pair. For a solution containing ions *A* and *B* with charges −1 and −2, respectively, their contribution to the charge capacity is then given by equation (2.2).2.2
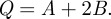


Solving for *A* and *B* gives the number of moles of each anion that is incorporated per gram of sample, as given by equations (2.3) and (2.4).2.3
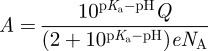
and2.4
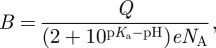
where *N*_A_ is Avogadro's number and *e* is the charge of an electron. The total ion exchange capacity, C (mol g^−1^), is then given by equation (2.5).2.5
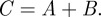


The different p*K*_a_ values were retrieved from SI chemical data [33]. As follows from equation (2.1), both oxalate solutions contained only (COO^−^)_2_, the isotonic phosphate solution contained only 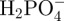 while the 0.10 M phosphate solution contained both 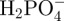 and 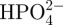, in significant amounts.

### 2.4. Blood compatibility study

#### 2.4.1. Blood sampling

Fresh human blood from 10 healthy donors was collected in heparin-coated 50 ml Falcon tubes (Becton–Dickinson, USA) containing soluble heparin (Leo Pharma), giving a final concentration of 1.5 IU heparin ml^−1^.

#### 2.4.2. The slide chamber model

The slide chamber previously described by Hong *et al*. [34] was used for these experiments. The device is manufactured from polymethylmethacrylate and consists of two wells that can hold a maximum volume of 1.6 ml each. After heparin coating, each well was filled with 1.4 ml of blood (1 ml of blood was also collected in Eppendorf tubes containing ethylenediaminetetraacetic acid (EDTA)–K_3_ or heparin; the latter samples were later used as 0 min controls). The composite membranes (previously soaked in saline solution) were then placed so as to cover the wells (as ‘lids’), thus two circular chambers were created. The slide chambers were rotated vertically at 22 rpm for 60 min in a 37°C water bath. After incubation, 0.6 ml of blood from each chamber was removed and mixed with EDTA–K_3_ or heparin, giving a final concentration of 10 mM or 10 IU ml^−1^, respectively. The EDTA samples (including the 0 min controls) were analysed for platelet numbers on a Coulter Ac·T diff haematology analyser (Coulter Corporation, Miami, FL, USA). Thereafter, the samples were centrifuged at 3000 *g* for 10 min at 4°C, and the plasma was collected and stored at −70°C for future analysis. The heparin-treated blood samples were analysed by flow cytometry. The membranes were fixed and dehydrated prior to the scanning electron microscopy (SEM) studies.

Three different types of commercially available haemodialysis membrane materials were also included in the blood compatibility study: unmodified regenerated cellulose (RC), modified cellulose and a synthetic polymer. Thus, the following materials were used as references: RC (Sartorius Stedim Biotech GmbH, Goettingen, Germany), cellulose acetate (CA; Sartorius Stedim Biotech) and polysulphone (PS; Pall Life Science, USA), all membranes had pore sizes of 0.20 and 0.45 µm. All materials were run in duplicate. Although unmodified RC is rarely used today in haemodialysis, it is a valuable reference material for comparative studies in the context of the present work.

#### 2.4.3. Enzyme immunoassays

PBS containing 1 per cent (w/v) bovine serum albumin (BSA; Sigma-Aldrich), 0.1 per cent Tween 20 (Sigma-Aldrich) and 10 mM EDTA was used as the working buffer, while PBS containing 0.1 per cent Tween 20 served as the washing buffer.

*Detection of C3a*. Plasma samples diluted 1/1000 or 1/5000 were incubated in wells coated with monoclonal antibody 4SD17.3 (capture antibody). C3a was detected with biotinylated anti-C3a antibody (Dako, Glostrup, Denmark) followed by horse radish peroxidase (HRP)-conjugated streptavidin (GE Healthcare, Uppsala, Sweden) [35]. Zymosan-activated serum, calibrated against a solution of purified C3a, served as a standard. The values were given in ng ml^−1^.

*Detection of sC5b-9.* sC5b-9 was measured using a modification of the method described by Mollnes *et al*. [36]. Plasma samples were diluted 1/3 and added to microtitre plates coated with antineoC9 monoclonal antibody. sC5b-9 was detected by polyclonal anti-C5 antibodies diluted 1/500 (Dako, Glostrup, Denmark), followed by HRP-conjugated anti-rabbit immunoglobulin diluted 1/500 (Dako, Glostrup, Denmark). Zymosan activated serum containing 40000 AU ml^−1^ served as standard. The values were presented as AU ml^−1^.

*Detection of thrombin–antithrombin complex* (*TAT*). Microtitre plates were coated with anti-human thrombin antibody (Enzyme Research Laboratories, South Bend, IN, USA) diluted 1/125. HRP-coupled anti-human AT antibody (Enzyme Research Laboratories) diluted 1/125 was used for detection. Pooled human serum diluted in normal citrate–phosphate–dextrose plasma was used as a standard. The values were presented as µg l^−1^.

#### 2.4.4. Flow cytometry

Samples for flow cytometry were collected in heparin (10 IU ml^−1^) and treated with human immunoglobulin (Beriglobin, ZLB Behring GmbH, Marburg, Germany), 4 mg ml^−1^, to block Fc receptors. Staining was performed by adding 20 µl of antibody to 100 µl of sample. After incubation at 4°C for 30 min, the samples were treated with Erythrolyse red blood cell lysing buffer (AbD Serotec, Oxford, UK) for 10 min. After centrifugation (400 *g*, 5 min), cell pellets were resuspended in PBS containing 1 per cent BSA and again centrifuged for 5 min at 400 *g.* Finally, the cells were resuspended in PBS and analysed using a BD LSRII SORP (BD Biosciences, CA, USA) flow cytometer. Monocytes and neutrophils were identified by their distinct patterns on forward scatter and side scatter and by appropriate surface markers. For monocyte identification, monoclonal FITC-conjugated anti-CD14 was used, while for neutrophil identification monoclonal allophycocyanin (APC)-conjugated anti-CD15 was applied. Monoclonal PE-conjugated anti-CD11b was used to evaluate the upregulation of CD11b expression on monocytes and neutrophils.

The antibodies for negative controls were mouse APC IgM kappa isotype control, PE mouse IgG1 kappa isotype control and FITC mouse IgG2b kappa isotype control. All antibodies were supplied by BD Biosciences.

The setting of flow cytometry compensation was performed using the AbC anti-mouse bead kit (Invitrogen, UK). AbC capture beads were single stained with the same fluorochrome-labelled antibodies used for cell staining, following the manufacturer's recommendations.

Data were analysed using a BD FACSDiva software (BD Biosciences). The mean fluorescence intensity of the staining of PE-conjugated anti-CD11b on monocytes and neutrophils was calculated by the software.

#### 2.4.5. Scanning electron microscopy

After blood contact, the composite membranes were washed with HBSS, fixed with 1.5 per cent (v/v) glutaraldehyde in HBSS, dehydrated through a series of ethanol concentrations (25, 50, 70, 80, 90 and 100% (v/v)), and followed by incubations with haexamethyldisilazane (HMDS) solutions (HMDS : ethanol 1 : 2, HMDS : ethanol 2 : 1 and 100% HMDS). Finally, the dehydrated samples were allowed to air-dry and were then kept in a desiccator prior to SEM analysis using a Leo 1550 SEM instrument (Zeiss, Germany).

#### 2.4.6. Statistical analysis

Non-parametric statistical analyses were performed using IBM SPSS Statistics v. 19. Data were statistically evaluated by independent samples median tests with pairwise comparisons of groups, adjusted for multiple comparisons. Samples were considered statistically different at *p* < 0.05.

## 3. Results and discussion

### 3.1. Heparinization

**Figure 1.**
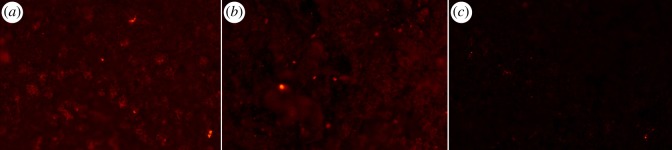
Heparin coating on the PPy–cellulose membranes stained with avidin–Texas Red observed under fluorescence microscopy. Panels show representative images of (*a*) heparinized PPy–cellulose, (*b*) heparinized PPy–cellulose after reduction–oxidation cycles, (*c*) non-heparinized PPy–cellulose membrane. Note the intense red staining distributed all over the surface of the heparinized samples (*a,b*). (Online version in colour.)

Modification of biomaterial surfaces with heparin is a well-established method to improve blood compatibility [29,37–39]. It is important to ensure that the AT binding sequence of heparin is left intact after heparin has been immobilized on the surface, and to control the stability of the heparin coating. The release of heparin during haemodialysis is particularly undesirable because a large amount of heparin may enter the blood circulation and this can increase the risk of abnormal haemorrhage or lead to heparin-induced thrombocytopaenia [40]. As shown in table 1, heparin was successfully immobilized on the composite surface, exhibiting an AT binding capacity of 9 ± 1 pmol cm^−2^. This value was statistically significant different from the negative control (non-heparinized composite), which showed 2 ± 1 pmol cm^−2^ owing to unspecific binding. The heparin coating applied to the composite membranes is due to electrostatic interactions. The heparin conjugate binds strongly to the cationic surface of the composite (oxidized PPy) as a result of the multiplicity of anionic groups on the heparin conjugate. When PPy is subjected to reduction and oxidation cycles, a certain loss of heparin could be expected because the reduction of the conductive polymer renders the material neutral in terms of charge. However, the macromolecular characteristics of the heparin conjugate, together with the presence of the polyamine and a second layer of the heparin conjugate, seems to prevent depletion of the heparin coating after the cyclic reduction–oxidation treatment, as indicated by the AT binding capacity of such sample (9 ± 1 pmol cm^−2^). Fluorescence microscopic images of the heparinized composite indicated the presence of the heparin coating on the material surface (figure 1*a*). The heparin layer remained on the membrane surface after repeated reduction–oxidation cycles of the heparinized composite (figure 1*b*). The image in figure 1*c* showed the absence of non-specific avidin-binding or autofluorescence of the composite surface because no prominent florescence signal could be detected.

**Table 1.** Evaluation of heparin coating on PPy–cellulose composites.

	AT binding capacity (pmol cm^−2^)^a^
heparinized composite	9 ± 1
heparinized composite after redox cycle	9 ± 1
heparinized composite after steam sterilization	8 ± 2
non-heparinized composite	2 ± 1

^a^Data represent mean value ± s.e. of the mean, *n* = 6.

The effect of steam sterilization on the heparin coating was also studied by evaluating the AT binding capacity of the sterilized heparin-coated membrane. The results indicated that the active properties of the heparin layer were unaffected by the autoclave process, because the AT binding capacity was found to be 8 ± 2 pmol cm^−2^ and not significantly different form the value found for the heparinized sample before steam sterilization (*p* > 0.05; table 1).

Other authors [41,42] have successfully modified PPy surfaces with heparin, with the aims of improving blood compatibility and stimulating endothelial cell growth. Li *et al*. [41] claimed that covalent bonding of heparin is the most effective method to heparin coat PPy. The authors mentioned that the main disadvantage of ionic immobilization of heparin onto PPy was the loss of heparin over time, while electrochemical entrapment of heparin could lead to reduced bioactivity of the entrapped heparin [41]. Nevertheless, thanks to the properties of the Corline heparin conjugate, we have shown that it is possible to immobilize heparin through ionic interactions onto PPy with the method described here, and that this gives rise to a stable and active heparin coating.

### 3.2. Feasibility study

The PPy–cellulose composite is a material combining the porous structure and large surface area of *Cladophora* cellulose [43] with the potential-controlled ion exchange capabilities of PPy [44]. Thus, with a PPy–cellulose composite in a haemodialysis setting, in addition to passive diffusion and ultrafiltration through the porous structure of the cellulose, solutes can be extracted by a potential-controlled ion exchange mechanism. To the authors' knowledge, this is the first study to investigate the ion exchange properties of a PPy–cellulose composite with respect to removal of specific uraemic toxins. Phosphate and oxalate were chosen as model solutes for testing the material's capability to extract solutes relevant in haemodialysis. The removal of phosphate in patients undergoing dialysis is insufficient, resulting in hyperphosphataemia. In fact, the average blood serum concentrations of phosphate has been found to be 6.2 mg dl^−1^ in end-stage renal disease (ESRD) patients that have undergone dialysis for at least 1 year [45], while the normal range for healthy individuals is 2.6–4.5 mg dl^−1^ [46]. Serum phosphate concentrations of 5 mg dl^−1^ or higher are related to an increased mortality risk [47]. Elevated oxalate concentrations in the blood (hyperoxalaemia) can lead to deposition of calcium oxalate in various tissues, including the heart [48]. In ESRD patients, it has been found that the average uraemic concentration is 4.9 ± 1.4 mg l^−1^, whereas the normal concentration is 0.3 ± 0.1 mg l^−1^ [49,50]. The ratio between the average uraemic and normal concentration was found to be among the 20 highest scoring solutes out of 45, highlighting oxalate as one of several solutes associated with uraemic toxicity [50].

**Figure 2.**
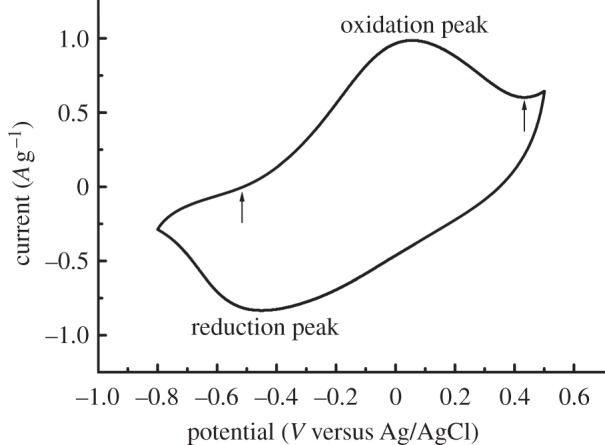
A representative cyclic voltammogram of the 10th cycle of a heparinized sample in an isotonic oxalate solution (0.12 M, pH 7.3) showing oxidation and reduction peaks corresponding to the incorporation and expulsion, respectively, of oxalate ions by the PPy–cellulose material. The arrows indicate the positions of the first point of positive charge and the local minima following the oxidation peak, which were used as limits for calculations of ion exchange capacities.

The capacity of the novel material to extract phosphate and oxalate ions was studied by CV-cycling of the composite samples in different phosphate and oxalate solutions. A representative cyclic voltammogram, resulting from CV-cycling of a heparinized sample in an isotonic oxalate solution (0.12 M, pH 7.3), is shown in figure 2. Notable features include the oxidation peak situated at approximately 0.05 V versus Ag/AgCl and the reduction peak located around −0.45 V versus Ag/AgCl. The peaks show that the PPy layer is indeed oxidized and reduced and, as a result, oxalate ions move in and out of the material, respectively. We cannot, however, rule out completely that some cations participate in the oxidation and reduction process. Similar voltammograms were obtained with heparinized and non-heparinized samples in isotonic or 0.10 M solutions of phosphate or oxalate, and only minor differences were observed between the electrolyte solutions.

**Figure 3.**
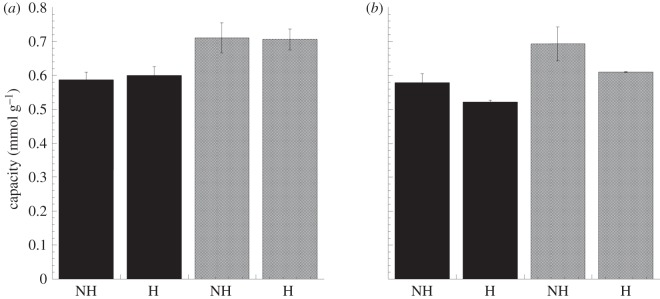
Ion exchange capacities calculated from the voltammograms, taking into account the distribution of ions in the solution at the corresponding pH for non-heparinized (NH) and heparinized samples (H). Panels show capacities in (*a*) 0.10 M (black bars; pH 7.4) and isotonic (grey bars; 0.15 M, pH 4.4) phosphate solutions and (*b*) 0.10 M (black bars; pH 7.4) and isotonic (grey bars; 0.12 M, pH 7.3) oxalate solutions. No significant differences in capacity were observed between non-heparinized and heparinized samples. The values are expressed as the mean ± s.e. of the mean, *n* = 3.

The extraction capacity of the material with respect to the studied ions was calculated based on the oxidation charge obtained from the CVs (figure 3), assuming no contribution from cation movements. It should be pointed out that the capacity of the material is a function of the conductivity and concentration of the electrolyte solution [20,51]. Thus, the calculated capacity does not necessarily reflect the true capacity in a haemodialysis application. However, the capacities are useful for comparing the extraction capabilities of heparinized and non-heparinized samples. No statistically significant capacity difference (*p* > 0.05) was observed between non-heparinized and heparinized samples cycled in a 0.10 M phosphate solution (pH 7.4; 587 ± 23 and 600 ± 26 μmol g^−1^, respectively) or an isotonic phosphate solution (711 ± 44 and 706 ± 31 μmol g^−1^, respectively). There was also no significant difference (*p* > 0.05) between the non-heparinized and heparinized samples when oxalate was extracted from the 0.10 M solution (pH 7.4; 579 ± 26 and 523 ± 5 μmol g^−1^, respectively) or from the isotonic solution (692 ± 50 and 610 ± 1 μmol g^−1^, respectively). These results demonstrate that it is possible to extract phosphate and oxalate ions from solutions with physiological pH and the same tonicity as blood. It should be noted that the data in this study are solely based on the extracted charges and that in order to get the exact number of extracted anions of each type, one needs to directly measure the amounts of anions actually extracted. The key point is, however, that heparinizing the PPy–cellulose composite has no effect on the material's extraction capacity.

The feasibility study illustrates that heparinized PPy–cellulose composites may possibly be used as a potential-controlled uraemic toxin extraction membrane, where solutes are removed also by extraction into the material. Further investigations into the applicability of the PPy–cellulose material will include physiological solute concentrations, mixed solvents, whole blood and eventually flowing blood. Moreover, investigations related to diffusion and ultrafiltration of non-ionic molecules through the porous matrix are currently underway. It should be mentioned that the ability of PPy to extract molecules from a blood flow has been demonstrated recently, where a PPy-based *in vivo* microextraction technique was used to extract pharmaceutical substances [52]. Furthermore, the material's capability of releasing solutes in response to an electrical stimulus should be explored because this property provides a possibility of designing new haemodialysis systems that could mimic the secretion function of the kidneys.

### 3.3. Blood compatibility study

The *in vitro* model used in this work makes it possible to analyse both the biomaterial surface in terms of cell adhesion and the cellular and molecular events that take place in the fluidic phase. Blood was collected in tubes containing soluble heparin at a final concentration of 1.5 IU ml^−1^. This heparin concentration simulates that in clinical dialysis and reflects appropriate anticoagulation, e.g. minimize changes in coagulation parameters while avoiding total depletion of platelets [24]. Three groups of haemodialysis membranes can be differentiated according to the raw materials used to manufacture them: unmodified RC, substituted cellulose and synthetic polymers. One membrane of each group was included in this study to compare their *in vitro* haemocompatibility with the performance of the novel PPy–cellulose haemodialysis membrane. Pore sizes of high cut-off membranes, typically larger than 200 nm, were selected [1]. The composite material has a broad pore size distribution in the mesopore range, i.e. between 2 and 100 nm, both in its heparinized and non-heparinized form as evidenced from the density function theory (DFT) analysis of N_2_ sorption isotherms (see electronic supplementary material, figure S1). It is seen from the DFT analysis that the pore size distribution in the samples is markedly skewed towards larger pore sizes. The latter is in line with the high-resolution SEM micrographs (see electronic supplementary material, figure S2), in which pores having widths of up to around 200 nm are clearly visible. It should be pointed out that no efforts have yet been made to obtain a narrow pore size distribution in the composite around a specific cut-off value, and this issue will be addressed in future work.

Haemocompatibility was assessed by determining the thrombogenic properties of the dialysis membranes, i.e. evaluation of platelet number and adhesion and the formation of TAT complexes, together with the study of complement activation and the upregulation of CD11b receptor expressed on neutrophils and monocytes.

#### 3.3.1. Thrombogenic properties

**Figure 4.**
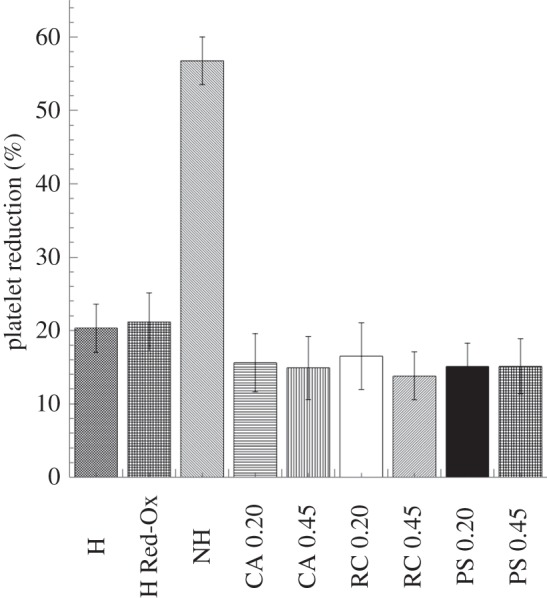
Platelet counts performed after whole-blood incubation with the studied materials (soluble heparin concentration 1.5 IU ml^−1^). The reduction in platelet number is expressed as percentage of the values obtained for the 0 min samples. A significant improvement in platelet reduction is achieved by heparin coating the composite material. Results also indicated the stability of the heparin coating when the heparinized composite is repeatedly reduced and oxidized. The values represent the mean ± s.e. of the mean from experiments using blood from 10 different donors. (H, heparinized composite; H Red-Ox, heparinized composite after reduction–oxidation cycles; NH, non-heparinized composite; CA, cellulose acetate; RC, regenerated cellulose; PS polysulphone, 0.2 represents 0.2 µm pore size and 0.45 indicates membranes with 0.45 µm pore sizes).

**Figure 5.**
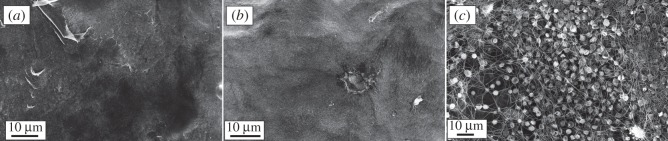
Representative scanning electron micrographs of PPy–cellulose membranes after 60 min incubation with whole blood (soluble heparin concentration 1.5 IU ml^−1^). The heparinized composites (before and after reduction–oxidation cycles, (*a*) and (*b*), respectively), showed minimal cell adhesion on the surfaces, while the non-heparinized composite presented a large number of adherent platelets and a mesh of fibrils (*c*).

Platelet counts were performed after blood contact with the studied materials and the results were expressed as percentage of the values obtained for the initial blood sample (0 min control; figure 4). The results indicated a marked reduction on platelet number when blood was in contact with the non-heparinized composite material. However, the heparin coating was very effective in improving blood compatibility in terms of reduction in platelet number (57 ± 3% versus 20 ± 3%). The percentage of platelet reduction observed with the heparin-coated membranes was comparable with the values obtained with the reference materials, i.e. no statistically significant difference was found between the reference materials and the heparinized composite. The comparison between the heparinized PPy–cellulose membrane before and after repeated reduction and oxidation cycles confirmed the stability of the heparin coating, because no significant difference in platelet reduction was found between these samples. Platelets are thought to become activated when they come in contact with an artificial surface, and their number decreases as they adhere to the material surface and aggregate. When the heparin-coated composite surface was studied using SEM, the images confirmed the poor platelet adhesion on the material surface (figure 5*a*). The same pattern was observed on the heparinized sample after reduction and oxidation cycles (figure 5*b*). However, SEM micrographs of the non-heparinized composites showed a large number of platelets and a mesh of fibrils (figure 5*c*).

**Figure 6.**
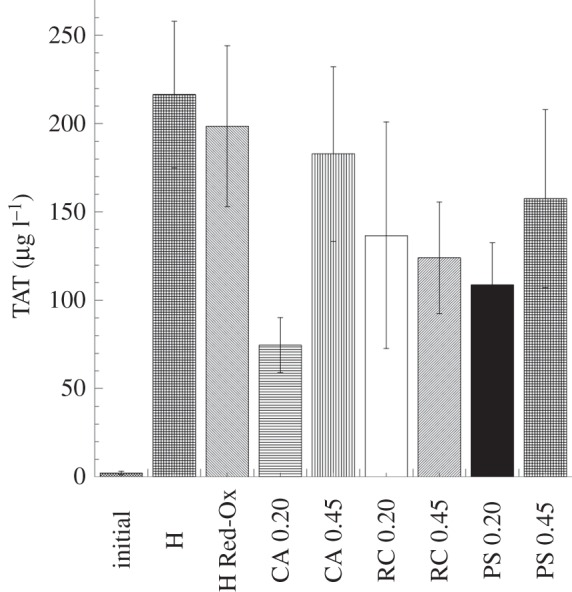
Thrombin–antithrombin complex (TAT) levels detected in the fluid phase after whole-blood (soluble heparin concentration 1.5 IU ml^−1^) contact with the heparinized PPy–cellulose membranes and the reference materials. The level of TAT complex generated by the non-heparinized PPy–cellulose membrane was (10 670 ± 1269 µg l^−1^) and significantly higher than the heparinized composites and the reference materials. This value was omitted in the figure for clarity. No significant difference was found between the heparinized composites and the reference materials. The values represent the mean ± s.e. of the mean from experiments using blood from 10 different donors. (H, heparinized composite; H Red-Ox, heparinized composite after reduction–oxidation cycles; CA, cellulose acetate; RC, regenerated cellulose; PS polysulphone, 0.2 represents 0.2 µm pore size and 0.45 indicates membranes with 0.45 µm pore sizes.)

The formation of thrombin was followed by measuring the TAT levels after blood contact with the studied materials, and the results showed that the thrombogenic properties of the heparinized composite were comparable with those of the reference materials (figure 6), while the non-heparinized material showed significantly higher thrombin generation. The level of TAT complex generated by the non-heparinized PPy–cellulose membrane was (10 670 ± 1269 µg l^−1^).

High platelet consumption can be related to the parallel thrombin generation because thrombin is a platelet activator. In addition, activated platelets induce thrombosis by secretion of bulk phase agonists, acceleration of thrombin formation and via fibrinogen-mediated platelet–platelet aggregation [21,53]. The non-coated composite material was found to be highly thrombogenic as reflected by the high reduction in platelet count, large platelet adhesion to the material surface and elevated levels of TAT. However, when the composite surfaces were coated with heparin conjugate, the formation of thrombin was significantly reduced and so was the adhesion of platelets to the biomaterial surface. Soluble heparin downregulates coagulation by binding to AT and turning it into a more active state, increasing the ability to inhibit different coagulation factors by 1000-fold [54]. The accepted theory behind the use of heparin coatings is that downregulation of coagulation is achieved by binding the regulator AT [22]. Nilsson *et al*. [55] proposed that the effect of heparin coating is that it turns the biomaterial surfaces into surfaces totally devoid of adsorbed host cells and platelets after contact with blood. The latter authors suggested that plasma protein binding to the heparin-coated surface is due to both non-specific and specific interactions and that the binding does not significantly change the conformation of the adsorbed proteins, which, hence, are not activated. In the case of platelet adhesion and subsequent activation by artificial surfaces, the conformation of adsorbed fibrinogen plays an important role [56]. Therefore, it can be speculated that the heparin coating not only suppresses the thrombin formation by its high AT binding capacity but also decreases platelet adhesion and activation because adsorbed fibrinogen on the heparin surface does not adopt the right conformation, i.e. does not expose platelet-binding sites.

#### 3.3.2. Complement activation

**Figure 7.**
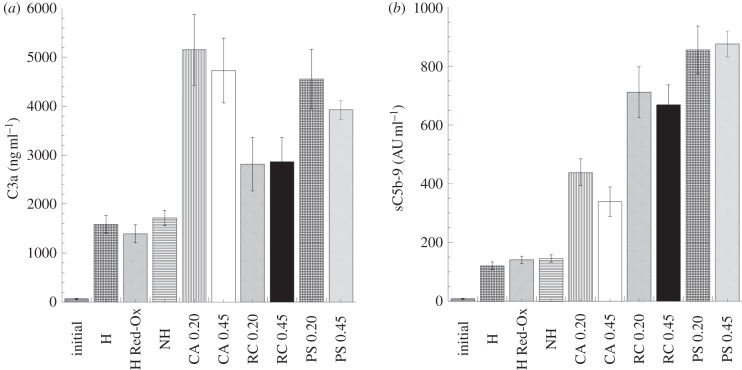
Complement activating properties of the PPy–cellulose composites and the reference materials after 60 min whole-blood incubation (soluble heparin concentration 1.5 IU ml^−1^). The novel PPy–cellulose haemodialysis membrane showed significant lower levels of C3a (*a*) than the commercially available membranes CA and significant lower levels of sC5b-9 (*b*) than the reference membranes CA, RC and PS. The values represent the mean ± s.e. of the mean from experiments using blood from 10 different donors. (H, heparinized composite; H Red-Ox, heparinized composite after reduction–oxidation cycles; NH, non-heparinized composite; CA, cellulose acetate; RC, regenerated cellulose; PS polysulphone, 0.2 represents 0.2 µm pore size and 0.45 indicates membranes with 0.45 µm pore sizes.)

Complement activation products C3a, C4a and C5a are used as candidates to evaluate complement activation in plasma samples. The C3a level is usually chosen as an indicator of total complement activation. C5a is the most potent anaphylatoxin but has the disadvantage of rapidly being coupled to its receptor. However, measuring of sC5b-9 is an excellent way to quantify terminal complement activation and indirectly assess C5a levels [57]. The novel PPy–cellulose haemodialysis membrane showed significant lower levels of C3a than the commercially available membranes CA (figure 7*a*). When measuring the levels of sC5b-9, we found that the composite membrane generated significant lower levels than the reference membranes CA, RC and PS (figure 7*b*). The presence of PPy coating on the cellulose fibres could explain the lower complement activation as it covers the OH groups on the cellulose. The significantly higher complement activation found for pure *Cladophora* cellulose/MFC membranes supports this hypothesis (C3a = 2370 ± 172 ng ml^−1^, sC5b-9 = 322 ± 49 AU ml^−1^).

We found unexpected high levels of C3a and sC5b-9 after blood contact with PS, a material commonly regarded as highly biocompatible. However, the difference in biocompatibility could be related to the models used in different studies (*in vitro* versus *in vivo* or *ex vivo*, semi-static model versus dynamic blood recirculation) and the material design (membrane versus hollow fibres) [58–61]. Moreover, when used in dialysers, PS is combined with hydrophilic additives or is processed with hydrophilic copolymers such as methallylsulphonate to compensate for its hydrophobicity [1]. Therefore, this difference in chemical composition could also contribute to the observed difference between the results of the present study and those of the others [58–61].

#### 3.3.3. Upregulation of CD11b on neutrophils and monocytes

An effect of complement activation is the upregulation of receptors on leucocytes such as CD11b/CD18 and CD35—which together with the down regulation of l-selectin—make the leucocytes very adhesive and prone to interact with platelets and endothelial cells [22]. This may play an important role in the pathophysiological changes that occur during a haemodialysis session.

**Figure 8.**
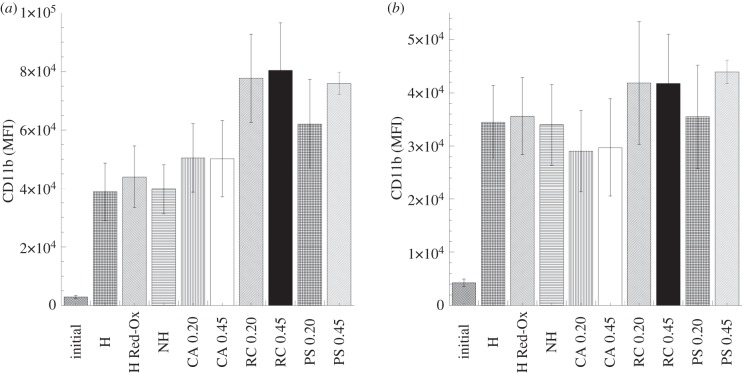
Modulation of neutrophil (*a*) and monocyte (*b*) CD11b expression after 60 min whole-blood incubation with the PPy–cellulose membranes and the reference materials (soluble heparin concentration 1.5 IU ml^−1^). The results are expressed as mean fluorescence intensity ± s.e. of the mean (*n* = 6). The evaluation of CD11b expression in neutrophils and monocytes did not show any significant difference between the studied membranes. (H, heparinized composite; H Red-Ox, heparinized composite after reduction–oxidation cycles; NH, non-heparinized composite; CA, cellulose acetate; RC, regenerated cellulose; PS polysulphone, 0.2 represents 0.2 µm pore size and 0.45 indicates membranes with 0.45 µm pore sizes.)

All studied materials showed an increase in CD11b expression, both in neutrophils and monocytes (figure 8*a* and *b*, respectively) after 60 min incubation with blood. However, such increase showed to be non-significant when statistically evaluated. The limitations of the non-parametric test, i.e. the use of ranks instead of continuous values, may mask the large differences that we actually see between the initial CD11b expression and the values obtained with the studied materials. The evaluation of CD11b expression in neutrophils and monocytes did not show any significant difference between the studied membranes (figure 8*a* and *b*, respectively). Thus, the differences found in complement activation between the studied materials were not reflected in the upregulation of CD11b expression. These results reflect the complexity of biochemical interactions at the blood–membrane interface and the importance of the evaluation of multiple parameters.

In summary, a set of haemocompatibility parameters were taken into account to evaluate the activation of blood after contact with the novel PPy–cellulose haemodialysis membrane. By including reference haemodialysis membrane materials, we were able to compare the biocompatible profile of the novel material with those of the well-established haemodialysis membranes. In terms of platelet adhesion and thrombin generation, the composite material proved to be highly thrombogenic, which—to a large extent—could be eliminated by applying a stable heparin coating. The heparinized composites behaved in the same way as the synthetic polymer PS. It is reasonable to assume that the heparin coating on the composite material can be further optimized leading to further improvement. When complement activation was evaluated in terms of sC5b-9 levels, the composites (uncoated and heparin-coated) were shown to be more biocompatible than the reference materials. In terms of upregulation of the adhesion receptor CD11b in neutrophils and monocytes, the reactivity of the composite membrane was similar to the reference materials.

The *in vitro* model presented in this work allowed for initial screening of the promising composite membrane material. Next steps will involve the use of *ex vivo* flow systems to measure the dynamic interactions of the components of blood with the novel haemodialysis membrane as well as to quantify the extraction of the anions by measuring the amounts of anions actually extracted.

## 4. Conclusions

The overall picture that emerges from this study is that the present PPy–cellulose composite is a promising haemodialysis membrane material, regarding both toxin removal performance and haemocompatibility. We have demonstrated the feasibility of using potential-controlled ion exchange properties of both non-heparinized and heparinized composites to remove small size uraemic toxins. Furthermore, no change in the ion exchange capacity could be detected after heparinizing the material. The thrombogenic properties of the composite were improved by applying a first generation of stable heparin coating on the material surface. Thus, in terms of platelet adhesion and thrombin generation, the heparinized composite is not different from the haemocompatible synthetic polymer PS. Moreover, the pro-inflammatory characteristics of the composite were, to some extent, superior to commercially available haemodialysis membranes.
